# Effect of large language model assistance on undergraduate art history question-answering performance: a randomized crossover pilot study

**DOI:** 10.3389/fpsyg.2026.1875025

**Published:** 2026-07-27

**Authors:** Yunting Zhang, Fan Zhang, Zili Zhang

**Affiliations:** 1 School of Fine Arts, Jining University, Jining, China; 2 Department of Visual Information Design, College of Creative Culture and Art, Namseoul University, Cheonan, Republic of Korea; 3 GE Healthcare Systems Trade Development (Shanghai) Co., Ltd., Shanghai, China; 4 School of Medicine, Shanghai Jiao Tong University, Shanghai, China

**Keywords:** art history, artificial intelligence, large language model, pilot study, randomized crossover study, undergraduate education

## Abstract

**Introduction:**

Large language models (LLMs) are increasingly used in higher education, yet empirical evidence for their effectiveness in art education remains scarce. This study aimed to evaluate whether LLM assistance could improve undergraduate art history question-answering performance and explanatory support.

**Methods:**

This study developed the Art History Theory Question Set (AHTQS), comprising 104 single-choice items with Bloom-level annotations, and benchmarked three LLMs (ChatGPT-4o, DeepSeek-V3, and Qwen2.5-Plus). DeepSeek-V3 showed the highest accuracy (96.2%) and lowest observed run-to-run variability and was selected for a randomized crossover pilot study with six undergraduates. The primary outcome was the change in examination accuracy from independent to LLM-assisted answering. A Likert-scale evaluation involving nine students and three instructors was also conducted to assess the clarity and coherence of LLM-generated explanations.

**Results:**

A one-sided Wilcoxon signed-rank test showed significant improvement with LLM support [*W*(6) = 21.0, *p* = 0.0156, *r* = 0.879], with median accuracy increasing from 43.3% to 93.3% (median gain = 42.3%). Five of six students showed higher accuracy under the LLM-assisted condition, and no clear evidence of a sequence or carryover effect was detected (Mann-Whitney U = 7.0, *p* = 0.3758). Domain-level analyses indicated significant gains in all four categories (*p* < 0.05). Error-frequency analysis further showed marked reductions in high-frequency mistakes. The Likert-scale evaluation indicated high perceived clarity and coherence of LLM explanations, with favorable but more cautious instructor ratings.

**Discussion:**

These pilot findings suggest that supervised LLM assistance may support art history question-answering and explanatory feedback. Future studies should validate these findings in larger cohorts, assess delayed learning retention, and examine open-ended, image-based, and higher-order art history tasks before curriculum-level implementation.

## Introduction

The effectiveness of theory-based instruction has become an important issue in higher education, while art history is a foundational theory course in art education. However, due to the complexity of its theoretical framework, the teaching of art history faces numerous practical challenges. On one hand, the course content is highly abstract and vast, encompassing multiple theoretical dimensions such as iconography, formalism, aesthetic philosophy, and social art history. It requires students to not only master extensive historical background, artists' styles, and ideological systems, but also to possess strong interdisciplinary integration and abstract comprehension skills (Ulbricht, 1998). On the other hand, resources for art history education in universities are relatively scarce. Common issues such as insufficient faculty, lack of teaching aids, and limited personalized course support hinder students, especially those in resource-limited areas, from accessing high-quality and personalized art history education resources (Budge, 2012). Traditional teaching in art history often relies on lectures and textbook reading; although this approach provides systematic content delivery, it depends heavily on teacher-led instruction and offers limited personalized support. As a result, students with different levels of prior knowledge may receive insufficient individualized support, which can limit deeper understanding and knowledge integration (Voskoglou and Salem, 2020). Consequently, many students generally struggle with understanding abstract theories, memorizing large amounts of information, and constructing systematic knowledge structures, which in turn affects their performance in art history learning and their ability to apply theoretical knowledge (Harris and Zucker, 2016).

With the rapid development of artificial intelligence (AI) technology, particularly advances in large language models (LLMs), the application of LLMs in education has become an active research area (Harris and Zucker, 2016; Jeon and Lee, 2023). Prior benchmark studies using examination-style questions have shown that LLMs can achieve competitive performance in structured educational tasks, including construction management (Genç, 2025a,b) and medical education (Wu et al., 2024), while prompting strategies such as self-consistency (Wang et al., 2022) and tree-of-thought (Yao et al., 2023) prompting may further improve reasoning performance (Durgun and Genç, 2025; Genç, 2025a).

Beyond benchmark evaluations, LLMs have also been tested in teaching and learning settings across engineering, medicine, and physics. For example, research by Pham et al. (2023) shows that the introduction of LLMs in university engineering education for assisted learning significantly enhances students' learning efficiency. Li et al. (2025) demonstrate that the introduction of LLMs can effectively improve performance in diabetes-related exams and the efficiency of primary care physician training, with ChatGPT-4.0 (Achiam et al., 2023) showing outstanding performance in both Chinese and English exams, suggesting potential value for medical education and clinical decision support. Latif et al. (2024) indicate that LLMs can provide timely feedback and explain domain knowledge in K-12 physics laboratory teaching, serving as a tool to enhance teaching efficiency and reduce teacher workload. Although these studies indicate the potential of LLMs for addressing complex teaching content and resource limitations, systematic evidence remains limited in humanities disciplines, especially art history.

To address this gap, this study combined domain-specific LLM benchmarking with a randomized crossover pilot experiment and post-task perceptual evaluation. The contributions of this study can be summarized as follows:

First, this study established the first standardized question bank in art history theory, providing a domain-specific benchmark for evaluating commonly used LLMs. Instructor responses were also included as an exploratory contextual reference for interpreting LLM performance on structured art history question-answering tasks.

Second, this study conducted a pilot study of LLM-assisted art history education, employing a randomized crossover design to provide preliminary evidence on whether LLM assistance can improve structured art history question-answering performance.

Third, this study used a Likert-scale survey to assess students' and instructors' perceived clarity and helpfulness of LLM-generated explanations, providing complementary evidence on perceived instructional utility.

Taken together, this study links domain-specific model benchmarking with learner-level evaluation, providing preliminary evidence for the use of LLMs in art history theory instruction. The findings may also inform future research on AI-supported teaching in the arts.

## Methods

**Figure 1:**
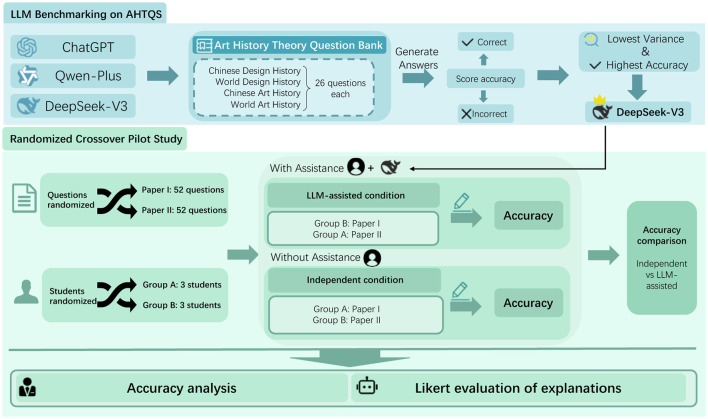
Study design flowchart.

The overall research process is illustrated in Figure 1.

### Construction of the Art History Theory Question Set

An Art History Theory Question Set (AHTQS) was developed and publicly released, to support automatic evaluations of LLM capabilities and real-world studies. The dataset is available for access and download at: https://github.com/ESCA001/LLM-Art-History-Benchmark. The question set consists entirely of four-option, single-choice items and is organized into four categories: Chinese Fine Art History, Global Fine Art History, Chinese Design History, and Global Design History. Each item is annotated with difficulty levels according to the 2001 revision of Bloom's Taxonomy (Forehand, 2010), covering six cognitive domains: remembering, understanding, applying, analyzing, evaluating, and creating, arranged from lower to higher levels of complexity.

All questions in AHTQS were sourced from authoritative textbooks, professional examination materials, and seminal scholarly works, such as *History of Chinese and Foreign Design, History of Modern World Design*, and *General History of World Art*. The initial answer key and Bloom-level annotations were prepared by an art history specialist with more than 10 years of experience (Yunting Zhang). To verify content accuracy, the answer key was cross-checked against the original source materials, and items with unclear wording, duplicated content, or ambiguous answers were revised before finalization. The final dataset was reviewed to ensure correctness, completeness, academic rigor, coverage of core knowledge points, and gradation of cognitive difficulty in undergraduate art history education.

To our knowledge, AHTQS is among the first publicly released benchmarks specifically designed for art-history theory question answering by LLMs, which may provide a foundational resource for advancing the application of AI technology in this domain.

### LLM selection and evaluation

Three widely used LLMs were selected for inclusion in the experiment, namely ChatGPT-4o (Achiam et al., 2023) (OpenAI, USA), DeepSeek-V3 (Liu A. et al., 2024) (DeepSeek, China), and Qwen2.5-Plus (Qwen et al., 2024) (Alibaba, China). These three models were selected because they were widely used, API-accessible, and high-performing models at the time of evaluation (White et al., 2024), and because they represented major model ecosystems relevant to both international and Chinese educational contexts. The model comparison was intended as a pragmatic selection step for the subsequent pilot study rather than an exhaustive ranking of all available LLMs.

The three LLMs were evaluated through Application Programming Interface (API)-based testing, with each model tested three times using the AHTQS under default parameter settings. All API calls were made through Alibaba Cloud Model Studio under default settings, with no manual adjustment of temperature, top-p, or other decoding parameters. The same prompt template (“The following is a single-choice question. Please select the correct answer based on the question:”) was used for all models, asking the model to select the best answer from the four options. The model with the highest accuracy and lowest performance variability was selected for the subsequent student pilot experiment. As an exploratory contextual comparison, two university instructors also completed AHTQS once. This comparison was intended to contextualize factual question-answering performance rather than to assess overall teaching quality or pedagogical superiority.

### Design of the real-world randomized crossover pilot study

To investigate the effectiveness of LLM-assisted learning in art history, the best-performing LLM was selected for a real-world randomized crossover pilot experiment. A total of six undergraduate students majoring in art-related disciplines were recruited. Their baseline knowledge levels were verified to ensure comparability across participants.

The AHTQS was randomly divided into two parts, Paper I, and Paper II, each containing an equal number of non-overlapping questions. Expert evaluation was conducted to confirm that the two parts were of comparable difficulty. Six students were randomly assigned into two groups (A and B). To minimize the potential influence of variations in exam difficulty and individual student ability on the results, a cross-over design was adopted: Group A completed Paper I independently and Paper II with LLM assistance, while Group B completed Paper II independently and Paper I with LLM assistance (see Figure 1). The comparison focused on changes in answer accuracy between independent responses and AI-assisted responses. Both groups completed the tests under comparable external conditions to control for environmental confounding factors.

In the LLM-assisted condition, students were allowed to submit the same question stem and answer options to LLM (through the web chat interface under default settings) and view the model-generated response, which included a recommended answer and explanatory text. Students then recorded their final answer after reviewing the model output. They were allowed to follow or reject the model recommendation. Therefore, the LLM-assisted condition was designed to evaluate assisted question-answering performance rather than unaided knowledge retention.

Given the exploratory nature of this randomized crossover pilot study, we specified two a priori hypotheses:

H1: Students' accuracy on art-history theory questions would be higher in the LLM-assisted condition than in the independent condition.

H2: The LLM-assisted condition would yield fewer recurrent high-frequency errors across participants than the independent condition.

### Evaluation of LLM-generated explanations

Upon completion of the tests, the ten questions with the highest error frequencies were selected, and the LLM was asked to generate explanatory answers for these items. The explanations were evaluated by students and instructors using five-point Likert-type scales, a commonly used format for assessing attitudes and perceptions (Likert, 1932). The evaluation dimensions were informed by technology acceptance and instructional feedback frameworks, emphasizing perceived ease of understanding, usefulness, clarity, relevance, and pedagogical value (Davis, 1989; Hattie and Timperley, 2007). Accordingly, the student scale included four indicators: Ease of Understanding, Logical Organization, Clarity of Expression, and Future Preference. The instructor scale included five indicators: Comprehensiveness, Terminology Accuracy, Case Relevance, Structural Rigor, and Instructional Value.

### Data analysis and visualization

A within-participant analysis was conducted using the Wilcoxon signed-rank test to assess accuracy changes from independent answering to LLM-assisted answering. Because the primary hypothesis was specified a priori and directional, namely that LLM-assisted accuracy would be higher than independent accuracy, one-sided tests were used for the prespecified paired comparisons. These results were interpreted within the exploratory context of a pilot study. The effect size ($r\;=\;Z/\sqrt{N}$) and a 95% bootstrap confidence interval (10,000 resamples) were additionally reported for the median paired gain. To examine potential sequence/carryover effects in the crossover design, for each student we computed the gain Δ = *accuracy*_*LLM*_−*accuracy*_*independent*_ and compared Δ between sequences (A → B vs. B → A) using the Mann–Whitney U test, summarizing effect magnitude with Cliff's δ. At the domain level (Chinese Design History, Chinese Fine Art History, Global Design History, Global Fine Art History), paired comparisons were performed for the four question categories using one-sided Wilcoxon signed-rank tests. At the individual level, per-student changes in accuracy were summarized descriptively; exploratory two-proportion z tests were also reported for transparency because Paper I and Paper II contained the same number of items and were reviewed for comparable difficulty. Domain-level and individual-level analyses were considered exploratory, no adjustment for multiple testing was applied, and the corresponding p-values were interpreted as nominal rather than confirmatory.

Likert-scale evaluations were summarized descriptively. Visualizations included bar charts (question-difficulty and Likert distributions) and box plots (paired outcomes). All analyses were performed in Python 3.8.18 using pandas 2.0.3, NumPy 1.24.3, SciPy 1.10.1, Matplotlib 3.7.3.

## Results

### Dataset construction

**Figure 2:**
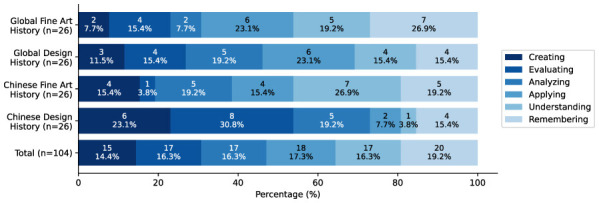
The distribution of difficulty levels in AHTQS according to the 2001 revision of Bloom's Taxonomy.

The AHTQS comprised a total of 104 single-choice questions, with 26 questions in each category. The distribution of difficulty levels across categories according to the 2001 revision of Bloom's Taxonomy is illustrated in Figure 2.

Overall, the questions were broadly distributed across the six cognitive levels, with “Remembering” (20/104, 19.2%) being the most frequent category and “Creating” (15/104, 14.4%) the least frequent. Notably, the Chinese Design History section exhibited a higher proportion of higher-order cognitive levels, including “Creating” and “Evaluating” (14/26, 53.8% in total), whereas the Global Fine Art History section contained a greater number of lower-level items, such as “Understanding” and “Remembering” (12/26, 46.2% in total). This pattern suggests a balanced yet slightly differentiated cognitive demand across the four domains.

### Evaluation of LLMs

Each of the three models (ChatGPT-4o, Qwen2.5-Plus, and DeepSeek-V3) was evaluated on the AHTQS three times, and both the average accuracy rate and the standard deviation of accuracy were calculated. The results are presented in Table 1.

**Table 1:** The accuracy rates of three LLMs and two art teachers in the AHTQS.

Responder	Accuracy (Mean ±SD %)				
	Total	Chinese Design History	Chinese Fine Art History	Global Design History	Global Fine Art History
ChatGPT-4o	92.6 ± 0.6%	94.9 ± 2.2%	88.5 ± 0.0%	92.3 ± 0.0%	94.9 ± 2.2%
Qwen2.5-Plus	95.2 ± 0.0%	96.2 ± 0.0%	92.3 ± 0.0%	96.2 ± 0.0%	96.2 ± 0.0%
DeepSeek-V3	96.2 ± 0.0%	96.2 ± 0.0%	92.3 ± 0.0%	96.2 ± 0.0%	100.0 ± 0.0%
Teacher 1^*^	72.1%	76.9%	80.8%	65.4%	65.4%
Teacher 2	67.3%	65.4%	73.1%	69.2%	61.5%

^*^The two teachers only answered AHTQS once, so there was no report of SD.

The results in Table 1 indicate that DeepSeek-V3 achieved the best overall performance among the three LLMs, with an average accuracy rate of 96.2%, outperforming Qwen2.5-Plus (95.2%) and ChatGPT-4o (92.6%). Moreover, DeepSeek-V3 attained the highest or joint-highest accuracy across all four question categories. In addition, it exhibited the lowest variability in accuracy, with no performance fluctuation, superior to ChatGPT-4o (SD = 0.6%) and equal to Qwen2.5-Plus. Considering both average accuracy and stability, DeepSeek-V3 was ultimately selected as the supporting model for the randomized crossover pilot study, to evaluate its effectiveness in assisting real-world teaching tasks.

To provide a contextual reference for factual question-answering performance on AHTQS, the responses of two university instructors were descriptively compared with those of the three LLMs. Both teachers were from the School of Fine Arts at Jining University and possessed more than 10 years of experience in art practice and teaching. As shown in Table 1, the average accuracy rates of all three LLMs were higher than those of the two teachers (72.1% and 67.3%, respectively), with higher accuracy across all categories. Notably, in the section of Global Fine Art History, where the teachers achieved their lowest scores (65.4% and 61.5%), DeepSeek-V3 attained a perfect accuracy of 100%. Conversely, although the lowest relative accuracy for DeepSeek-V3 was observed in Chinese Fine Art History (92.3%), this was still higher than the teachers' best-performing domain (80.8% and 73.1%, respectively). While variations in accuracy were observed across different subdomains, DeepSeek-V3 achieved higher factual accuracy than the two instructors in this limited structured benchmark, though this does not equate to pedagogical superiority. This finding suggests that DeepSeek-V3 may serve as a complementary instructional support tool in art history education.

### Real-world randomized crossover pilot study

The 104 single-choice questions were randomly divided into two test papers, Paper I, and Paper II, and the division was reviewed and confirmed by an expert in art (Yunting Zhang). As shown in Table 2, we did not detect evidence of a difference in the accuracy rates of the three LLMs between Paper I and Paper II (all *p* > 0.05), supporting the comparability of the two test papers.

**Table 2:** Comparison of the accuracy rates of the three LLM models on Question set part I and II.

LLM	Part I accuracy (%)	Part II accuracy (%)	*P*-value (paired t test)
ChatGPT-4o	84.62%	80.77%	0.6083
Qwen2.5-Plus	88.46%	86.54%	0.7695
DeepSeek-V3	94.23%	88.46%	0.3000

The pilot study enrolled six undergraduate students majoring in art-related disciplines. Their baseline data are presented in Table 3, including academic year, average grade, and grade in art history courses. No significant differences were observed in any of the three baseline measures between groups A and B (Mann-Whitney test, all *p* > 0.05).

**Table 3:** Baseline data of undergraduate students in groups A and B.

Group	Student No.	Average grade	Grade in art history course	Academic year
A	#1	87.6	86	Junior (3)
	#2	79.9	60	Junior (3)
	#3	87.2	83	Sophomore (2)
B	#4	87.5	66	Junior (3)
	#5	89.5	90	Freshman (1)
	#6	84.3	76	Junior (3)
*P*-value (Mann-Whitney test)		0.7000	>0.9999	>0.9999

**Figure 3:**
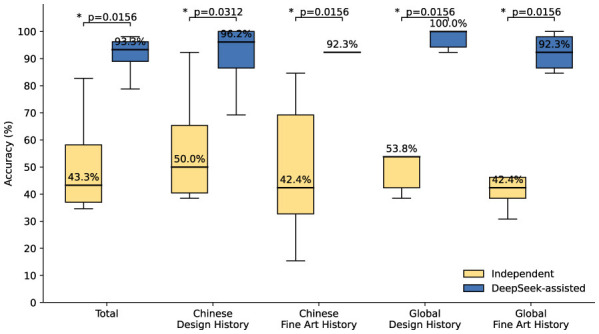
Comparison of the accuracy rates of six undergraduate students' independent responses and those assisted by DeepSeek-V3. *indicates a nominally statistically significant difference between the independent and DeepSeek-assisted conditions (one-sided Wilcoxon signed-rank test, *p* < 0.05).

The pilot study results demonstrated a significant improvement in students' art history examination performance following DeepSeek-V3 assistance (Wilcoxon signed-rank test, one-sided, W = 21.0, *n* = 6, *p* = 0.0156, *r* = 0.879). The median accuracy rate increased from 43.3% to 93.3%, corresponding to a median paired gain of 42.3% (95% bootstrap CI: 21.2%−59.6%; IQR: 38.0%−53.9%). Individual student gains on the AHTQS ranged from 5.8% to 61.5%, with exploratory two-proportion z tests showing consistent improvement patterns in five students (Table 4; one-sided two-proportion z-test; the remaining student *p* ≈ 0.20). Given the small number of students in each sequence, no clear evidence of a sequence or carryover effect was detected between the A → B and B → A sequences (median gains: 42.3% vs. 36.5%; Mann-Whitney U = 7.0, *p* = 0.3758; Cliff's δ = 0.556). Exploratory subgroup analyses across the four question categories showed nominally significant improvements in all four domains (Figure 3; all nominal *p* < 0.05, one-sided Wilcoxon signed-rank test).

**Table 4:** Individual accuracies and exploratory two-proportion z-test results on AHTQS.

Group	Student no.	Independent	LLM-assisted	Gain (%)	Z	One-sided *p*-value
A	#1	19/52, 36.5%	41/52, 78.8%	+42.3	4.367	6.31 × 10^−6^
	#2	25/52, 48.1%	47/52, 90.4%	+42.3	4.674	1.48 × 10^−6^
	#3	18/52, 34.6%	50/52, 96.2%	+61.5	6.596	2.12 × 10^−11^
B	#4	20/52, 38.5%	50/52, 96.2%	+57.7	6.271	1.79 × 10^−10^
	#5	43/52, 82.7%	46/52, 88.5%	+5.8	0.837	0.201
	#6	32/52, 61.5%	51/52, 98.1%	+36.5	4.641	1.73 × 10^−6^

The detailed error counts are presented in Table 5. The data reveal that during the independent answering phase without LLM assistance, among the 104 questions, 19 questions were answered incorrectly three times, 30 questions were answered incorrectly twice, and 33 questions were answered incorrectly once, while only 22 (21.2%) questions were answered correctly by all students. After the introduction of DeepSeek-V3 assistance, the accuracy improved significantly. Only 1 question was answered incorrectly three times, 3 questions were answered incorrectly twice, and 18 questions were answered incorrectly once. In contrast, 82 questions were answered correctly by all students, increasing the proportion of items answered correctly by all students to 78.8%, with an improvement of 57.7%. Moreover, the number of frequently mis-answered questions decreased markedly both overall and across the four subcategories. Notably, only one question from Chinese Fine Art History remained completely incorrect even with LLM assistance, underscoring the marked reduction of high-frequency errors facilitated by the model.

**Table 5:** Number of questions answered incorrectly by frequency (0–3).

Category	Answering method	Number of questions answered incorrectly			
		0	1	2	3
Total	Independent	22	33	30	19
	LLM-assisted	82	18	3	1
Chinese Design History	Independent	7	6	9	4
	LLM-assisted	19	6	1	0
Chinese Fine Art History	Independent	4	11	6	5
	LLM-assisted	23	2	0	1
Global Design History	Independent	5	9	10	2
	LLM-assisted	22	3	1	0
Global Fine Art History	Independent	6	7	5	8
	LLM-assisted	18	7	1	0

### Evaluation of LLM-generated explanations

**Figure 4:**
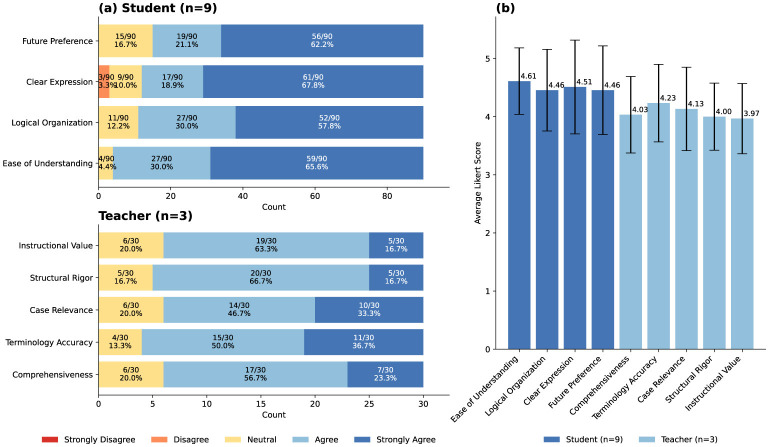
Student and instructor ratings of DeepSeek-V3 explanations for frequently missed questions. **(a)** Distribution of five-point Likert-scale ratings from students (*n* = 9) and instructors (*n* = 3) across the evaluation dimensions. **(b)** Mean Likert scores with error bars for each evaluation dimension among students and instructors.

Nine undergraduate students majoring in fine arts and three university teachers were recruited to evaluate the explanations generated by DeepSeek-V3 for ten frequently mistaken questions from AHTQS using five-point Likert scale. The distribution of scores is shown in Figure 4a. Among the student group, the proportion of “Strongly Agree” ratings was the highest across all four evaluation dimensions, ranging from 57.8% (Logical Organization) to 67.8% (Clear Expression). In contrast, among the teachers, the highest proportions were consistently “Agree” across all five dimensions, ranging from 46.7% (Case Relevance) to 66.7% (Structural Rigor). Median ratings were 5 across all student evaluation dimensions and 4 across all instructor evaluation dimensions. Both teachers and students rarely, if ever, selected “Strongly Disagree” or “Disagree.” By assigning numerical values from 1 to 5 to the five-point Likert scale (from lowest to highest), the average score for each evaluation dimension was calculated. The results are illustrated in Figure 4b. Among the student group, all four evaluation dimensions received mean scores of 4.46 or higher, with the highest reaching 4.61. In contrast, the teacher group's mean scores across all five dimensions were 4.23 or below, with the lowest being 3.97. While both groups gave generally favorable ratings, instructor ratings were numerically lower than student ratings across the evaluated dimensions. This pattern was interpreted descriptively because of the small number of instructors and because the student and instructor scales assessed related but non-identical dimensions.

## Discussion

### Principal findings

Emerging AI technologies, especially LLMs, show substantial promise for educational support, and this promise has already been demonstrated with rigorous experimental designs and statistical analyses in several STEM-adjacent domains, including engineering, medicine, and physics (Pham et al., 2023; Li et al., 2025; Latif et al., 2024). By contrast, art education has historically relied more heavily on arts-based and qualitative methodologies and diverse interpretive frameworks, resulting in a lack of empirical results from rigorous studies (Wan et al., 2018). Although AI is rapidly transforming creative fields and fine-arts workflows, scholarship on AI/LLM efficacy within art education remains relatively nascent and is often descriptive or exploratory rather than outcome-driven and statistically validated [Sáez-Velasco et al. (n.d.)]. This methodological gap has slowed the formal validation of LLM effectiveness in art education, even as broader higher-education surveys indicate substantial AI uptake among students and instructors at large.

To begin addressing this methodological gap, this study provides preliminary evidence that LLMs can provide substantial instructional support in art history theory education. Consistent with prior benchmark and prompt-engineering studies in educational contexts, the high AHTQS accuracy of DeepSeek-V3 suggests that LLMs can perform strongly on structured domain-specific question-answering tasks [Durgun and Genç, 2025; Genç, 2025a; Zong et al., 2026]. More importantly, the randomized crossover results extend this benchmark evidence by showing that LLM assistance may improve learner-level performance in art history theory education, particularly in reducing high-frequency errors. These findings suggest that LLM support may help reinforce foundational learning by clarifying difficult concepts, organizing knowledge, and supporting students' reasoning during question answering (Soverns-Reed, 2025).

Likert-scale evaluations suggested that students perceived the LLM-generated explanations as clear, coherent, and easy to understand, while instructors also recognized their pedagogical value but rated them more cautiously (Jeon and Lee, 2023). This pattern is consistent with prior studies showing that higher-education faculty often view generative AI as useful but remain concerned about accuracy, academic integrity, ethical use, and overreliance (Wang et al., 2024; Mamo et al., 2024). Therefore, instructor caution should be interpreted not as rejection of LLM-assisted teaching, but as a signal that classroom implementation requires supervision, clear usage guidelines, and discipline-specific validation.

Taken together, these results highlight the complementary role of LLMs in art history instruction. While they can effectively enhance knowledge reinforcement and personalized learning, they remain insufficient as substitutes for human educators, particularly in higher-order instructional tasks that require contextual interpretation, critical thinking, and interdisciplinary synthesis (Tan et al., 2025). The instructor comparison should therefore be understood only as a factual accuracy reference on multiple-choice items, not as an evaluation of teachers' broader pedagogical roles in explaining difficult ideas, guiding interpretation, and cultivating critical thinking. Therefore, LLMs should be strategically integrated into human-centered pedagogical frameworks that preserve teachers' guiding roles and emphasize the development of critical and reflective thinking (Raoulji, 2025). In practical terms, this integration requires well-designed safeguards, such as cognitive-forcing strategies and structured prompting methods, to prevent student overreliance and encourage critical evaluation of AI-generated content (Buçinca et al., 2021).

### Potential challenges and risk analysis

Despite the promising results, our study also surfaced important limitations and risks associated with LLM-assisted learning, which merit careful attention.

### Hallucination

It is widely acknowledged that the most critical issue in LLM applications is the presence of hallucination phenomena [i.e., generating plausible but untrue content (Liu F. et al., 2024)]. In our experiment, one particular AHTQS item, “What was the primary material used in China's earliest sculptural art?”, was mis-answered in all trials, even with LLM assistance. This case exemplifies how LLMs may occasionally generate responses that are semantically fluent and logically coherent yet factually inaccurate, even for foundational factual questions (Sriramanan et al., 2024). Such hallucination issues can potentially hinder students' knowledge construction by embedding misconceptions into their mental models (Elsayed, 2024). In art history especially, where precision and contextual nuance matter, such errors may be particularly problematic: learners may accept erroneous “facts” uncritically, compounding misunderstanding.

### Response variability and inconsistency

A second challenge is that repeated invocations of the same question may yield different answers. This variability reflects the probabilistic nature of LLMs. Because LLMs sample from probability distributions influenced by contextual prompt features and stochastic decoding strategies, they cannot guarantee identical outputs across iterations (Herrera-Poyatos et al., 2025). Although such variation does not typically undermine the overall quality of output (Shaier et al., 2024), repeated inconsistencies may exacerbate confusion regarding core concepts and ultimately diminish learners' confidence and judgment (Zhou et al., 2024). In practice, encountering inconsistent answers, even if both are reasonably phrased, may prompt students to doubt their own understanding or the reliability of the tool, inhibiting trust and learning stability.

### Implications and mitigation strategies

Given these risks, LLMs should be integrated into education cautiously. First, curriculum designers and instructors must remain vigilant: explicitly flag potential hallucination-prone content and encourage students to treat LLM outputs critically (e.g., cross-checking with authoritative sources). Second, implementing consistency-check prompts (asking the model to produce multiple answers or justify its output) can help detect unstable or suspect responses (Dhuliawala et al., 2023). Third, hybrid designs combining LLM output with retrieval-augmented fact checking may reduce ungrounded answers (Tonmoy et al., 2024). Finally, educating students about the probabilistic and stochastic nature of LLMs can foster metacognitive awareness: knowing that variability is expected may reduce confusion when discrepancies occur.

## Limitations and future directions

This study has several limitations, each of which suggests directions for future research. First, the pilot sample was small and drawn from art-related majors at a single institution, and the Likert-scale evaluation also involved a limited number of students and instructors. Future studies should include larger and more diverse cohorts across institutions, academic years, and disciplinary backgrounds. Second, the assessment relied mainly on single-choice questions and measured assisted question-answering performance rather than retained learning. Future work should incorporate open-ended responses, image-based judgments, argumentative analysis, and delayed post-tests without LLM support to determine whether LLM-assisted gains translate into durable knowledge retention. Third, although AHTQS was reviewed by a domain specialist and its answer key was checked against source materials, Bloom-level annotations were not independently rated by multiple experts. Future versions of AHTQS should involve multi-rater validation of answer keys and cognitive-level labels, with inter-rater reliability reported where applicable. Finally, the study focused on introductory undergraduate instruction and did not evaluate more advanced art history tasks, such as theoretical application, cross-cultural comparison, contextual interpretation, or critical writing. Future implementation studies should examine how LLMs can be integrated into instructional workflows for supervised explanation, personalized feedback, error attribution, and learning-path recommendation while maintaining teacher oversight and safeguards against overreliance.

## Conclusions

This randomized crossover pilot study suggests that LLM assistance can improve undergraduate students' performance on structured art history theory questions and reduce recurrent errors. Beyond these empirical findings, this study contributes to the literature by developing AHTQS as a publicly available benchmark for art history theory question answering and by extending LLM-assisted learning evaluation to a humanities discipline. Practically, the findings support the cautious use of LLMs as supervised instructional aids for concept explanation, formative practice, and individualized feedback in art history education. Given the small sample size and the focus on multiple-choice questions, future studies should evaluate larger and more diverse cohorts, open-ended and image-based tasks, longer-term learning outcomes, and implementation guidelines for responsible classroom integration.

## Data Availability

The datasets presented in this study can be found in online repositories. The names of the repository/repositories and accession number(s) can be found in the article/supplementary material.
